# Separation and Detection of *Escherichia coli* and *Saccharomyces cerevisiae* Using a Microfluidic Device Integrated with an Optical Fibre

**DOI:** 10.3390/bios9010040

**Published:** 2019-03-14

**Authors:** Mohd Firdaus Kamuri, Zurina Zainal Abidin, Mohd Hanif Yaacob, Mohd Nizar Hamidon, Nurul Amziah Md Yunus, Suryani Kamarudin

**Affiliations:** 1 Department of Chemical and Environmental Engineering, University Putra Malaysia, Selangor 43400, Malaysia; firdaus.kamuri@mara.gov.my (M.F.K.); suryani@upm.edu.my (S.K.); 2 Department of Computer and Communications Engineering, University Putra Malaysia, Selangor 43400, Malaysia; hanif.yaacob@gmail.com; 3 Department of Electrical and Electronic Engineering, University Putra Malaysia, Selangor 43400, Malaysia; mnh@upm.edu.my (M.N.H.); amziah@upm.edu.my (N.A.M.Y.)

**Keywords:** chip in a lab, dielectrophoretic, field flow fractionation, optical fibre, integrated

## Abstract

This paper describes the development of an integrated system using a dry film resistant (DFR) microfluidic channel consisting of pulsed field dielectrophoretic field-flow-fractionation (DEP-FFF) separation and optical detection. The prototype chip employs the pulse DEP-FFF concept to separate the cells (*Escherichia coli* and *Saccharomyces cerevisiae*) from a continuous flow, and the rate of release of the cells was measured. The separation experiments were conducted by changing the pulsing time over a pulsing time range of 2–24 s and a flow rate range of 1.2–9.6 μL min${}^{-1}$. The frequency and voltage were set to a constant value of 1MHz and 14Vpk-pk, respectively. After cell sorting, the particles pass the optical fibre, and the incident light is scattered (or absorbed), thus, reducing the intensity of the transmitted light. The change in light level is measured by a spectrophotometer and recorded as an absorbance spectrum. The results revealed that, generally, the flow rate and pulsing time influenced the separation of *E. coli* and *S. cerevisiae*. It was found that *E. coli* had the highest rate of release, followed by *S. cerevisiae*. In this investigation, the developed integrated chip-in-a lab has enabled two microorganisms of different cell dielectric properties and particle size to be separated and subsequently detected using unique optical properties. Optimum separation between these two microorganisms could be obtained using a longer pulsing time of 12 s and a faster flow rate of 9.6 μL min${}^{-1}$ at a constant frequency, voltage, and a low conductivity.

## 1. Introduction

Chip-in-a-lab commonly contains many important components including separation and sensing elements. According to a definition provided by [1,2], chip-in-a-lab requires the use of ancillary equipment and bulky supporting instrumentation such as fluidic pumps and high-current power supplies. While both chip-in-a-lab and lab-on-a-chip are categorised under micro total analysis systems, the former requires bulky equipment such as pumps while the latter uses the small size of the individual components in a chip. Many research reports on the applications of chip-in-a-lab were aimed at the separation of particle [3,4], bacteria [5,6] and cells [7,8].

The advantages of chip-in-a-lab over conventional identification are that the device can integrate and miniaturize sorting and detecting processes in a similar platform that can reduce labour, and lower volume and duration [9]. Furthermore, requiring lower volumes of reagents significantly lowers the cost, which is an important concern in clinical laboratories [10]. On the other hand, there is comprehensive research on the single system instead of the integrated system in chip-in-a-lab. This is due to the fact that it might be easier to fabricate a single system compared to an integrated system of chip-in-a-lab. However, surface modification is one of the main challenges in the fabrication of a chip-in-a-lab [11,12]. This study falls in the category of chip-in-a-lab.

Microfluidic is the manipulation of fluids at a microscale level and commonly uses electrokinetic and hydrodynamic forces to sort particles [13]. Dielectrophoresis is a branch of electrokinetics that uses microorganism/cell unique dielectric properties to yield different microorganism behaviour when subjected under non-uniform AC electric fields. On the other hand, field-flow fractionation is a separation principle that is based on the size of particles using drag flow [14]. When FFF is used in combination with DEP, the microorganism can be separated by means of different factors; such as diffusion, hydrodynamic, dielectric and other effects that eventually produces a net effect on the microorganism. This technique has a wide range of applications: it can enable efficient cell manipulation [15,16] and reduces the complexity of the LoC [17]. However, this approach is insensitive to the separation of two microorganisms with similar size and density [18,19]. Furthermore, dielectrophoresis commonly uses high frequency AC voltages which can produce Joule heating and damage the cell [20].

Meanwhile, optical techniques play a vital role in the chemical and biochemical analysis and thus, have a high possibility to be integrated in lab-on-a-chip microsystems [21,22]. In recent years, there has been an increasing interest in microsystems based on external light sources and photo-detectors [23]. A wide range of optical sensor systems have been employed for bacterial detection including high-index waveguide sensors [24], surface plasmon resonance sensors [25,26] and fibre optic techniques [27,28]. Optical methods have many advantages for a promising and reliable real-time bacterial detection. These advantages include their abilities to be miniaturised, flexibility for multiplexing [29] and producing rapid response times with high sensitivity for analyte evaluation [30]. The development of multi-analyte detection paves the way towards integrated biosensor design. High throughput analysis demands the detection of multiple analytes which suits very well with the optical method’s capabilities as mentioned earlier. Optical detection is preferred for robust and sensitive chip-in-a-lab because of the limitations of electrochemical and mechanical techniques. Previous development of chip-in-a-lab systems which contained electrochemical and optical based methods often suffered from drawbacks such as the complexity of integration and the lack of sensitivity [31,32]. However, on another note, others have reported that it can easily be miniaturized and incorporated into microfluidic systems using a simple design [33,34]. Zinoviev and co-workers showed that a microfluidic device based on integrated Bimodal Waveguides (BiMW) can be produced with a simplified fabrication process by neglecting the need for a reference arm [35].

This work aims to fabricate a chip-in-a-lab to produce a particle fractionation and detection system. The device employs an interdigitated microelectrode and embedded fibre optics for particle separation and detection. The device employs DEP-FFF and photonics technology for the fractionation and detection of microorganism inside a microfluidic device respectively. Furthermore, the functionality of the chip-in-a-lab was verified experimentally using *S. cerevisiae* and *E. coli*. In this study, the performance of the integrated device was characterized in terms of the comparison of the rate of release and the percentage of cumulative absorbance. The study offers some fresh insights into the potential of the chip-in-a-lab as a biosensor with a cheap and user-friendly device with a less complicated fabrication.

## 2. Materials and Methods

### 2.1. Preparation of Microorganism

*E. coli* (strain BL21) strains were grown overnight in Luria-Bertani (LB) Miller broth and on LB Miller agar at 37${}^{\circ }$ in a shaker at 150 rpm. After the inoculation process, the solution was transferred and centrifuged three times at 8000 rpm for 3 min using a high-speed centrifuge (Model D-37520 Osterode, Kendro Heraeus, Germany). The supernatant was removed and replaced with distilled water after each centrifugation process. The centrifugation process was necessary to remove unwanted particles and to obtain a low and stable value of low conductivity.

Dry yeast (*S. cerevisiae*) was used as the source of *S. cerevisiae* yeasts. An amount of 0.1 g of yeast powder was dissolved in 10 mL of distilled water (DI) water and kept in a water bath for 30 min to produce the live yeast solution. The live yeast solution was centrifuged at 8000 rpm for 3 min using a high-speed centrifuge. The supernatant was discharged and the pallets were resuspended by pipetting 10 mL of DI water. This procedure was repeated three times to eliminate unwanted particles and to reach a low and stable value of low conductivity. The number of cells was 2.25 ×$10^{8}$ cells/mL measured by a standard haemocytometer technique.

### 2.2. Design and Fabrication of the Chip-in-a-Lab

**Figure 1:**
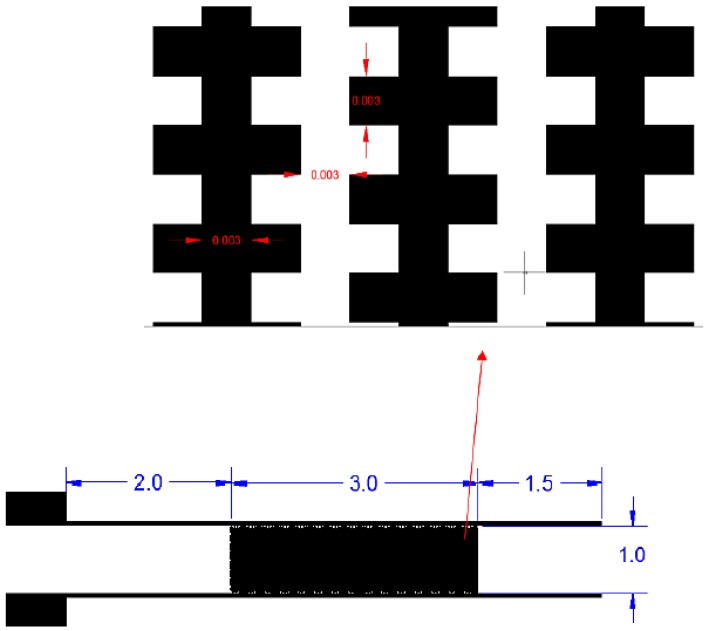
Interdigitated castellated microelectrode mask design with width and length of 1 cm and nearly 3 cm respectively.

The microelectrode system was constructed using photolithography fabrication techniques. All the equipment and facilities were located at Telekom Research and Development Sdn Bhd, Cyberjaya, Selangor, Malaysia. The microelectrode was designed using the AutoCAD 2007 program (Figure 1). The size of the interdigitated castellated microelectrode and the gap between the arrays of microelectrodes was designed to be 30 μm due to the limitation of the fabrication machine. Meanwhile, a microelectrode of 250 nm in height, 1 cm in width was formed by deposition of titanium (Ti) and gold (Au).

The interdigitated microelectrodes were fabricated on a glass substrate ( 24.0 mm × 60.0 mm × 3.0 mm). The commonly used AZ 1518 positive photoresist was used as the etching mask in this work. A standard photolithographic process was adopted to fabricate 30 μm sized microelectrodes.

**Figure 2:**
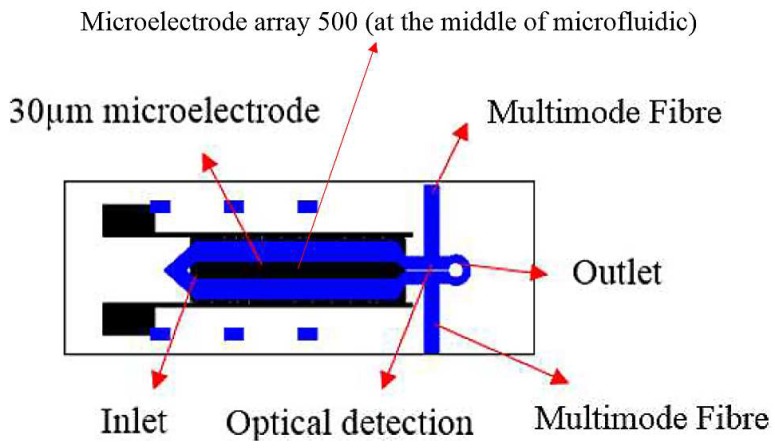
A schematic diagram of the integrated Lab on Chip system.

The microfluidic channel was fabricated on a glass substrate using dry film resist (DFR) (Ordyl Alpha 940) by soft lithography. The microfluidic fabrication was prepared according to the procedure used by [36,37]. For the patterning processes, microstructures were designed in the Autocad program and fabricated on a negative mask (Figure 2).

The design of the microfluidics was produced on DFR using transparencies printed by a high resolution printer as a photomask. The microfluidic channel was designed and fabricated on two glass substrates (one substrate had a microelectrode fabricated on it from the previous experiment) using a photolithography method. Based on the design, the inlet and outlet holes were drilled using a Dremel tool with a diamond drill bit. The design of the channel was divided into two parts; namely; separation and detection and contained one inlet and one outlet to allow the fluid to flow into and out of the channel.

### 2.3. Alignment of Multimode Fibre Pigtail for Optical Detection

In this work, the author used a ST multimode fibre pigtail as the transduction platform. The fibre core and cladding diameters were 62.5 μm and 125.0 μm respectively. The multimode pigtail was purchased from Ingellen Technology Co (Shenzhen, China). Initially, the multimode pigtail was cut using a fibre cleaver (FC-6RS, Sumitomo Electric, Osaka, Japan) in typically 5 cm lengths. Then, the thin plastic coatings of the bare fibres were stripped off with a fibre stripper. Once the cleaving process was finished, the fibres were cleaned with a Kimwipe soaked in ethanol. The fibres were then inserted and secured in placed on the DFR.

**Figure 3:**
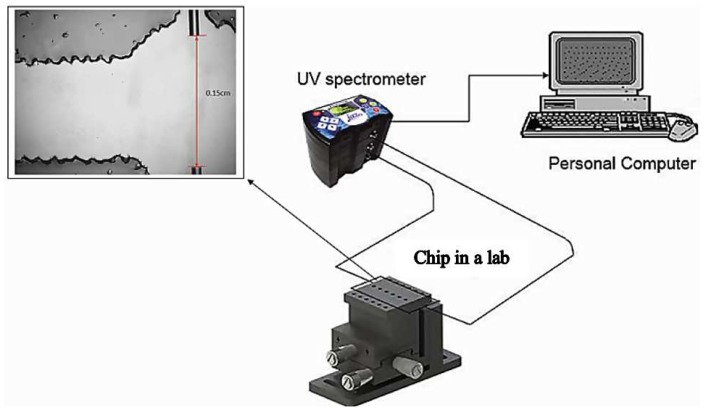
Fibre alignment setup using UV spectrometer and ball bearing vertical linear stages.

Fibre alignment was performed by measuring the transmittance output from the input multimode pigtail. Aligning the output multimode pigtail directly to the input multimode pigtail and bringing the two ends together as close as possible served as the base calibration factor for any power losses incurred by the multimode pigtail ends. The multimode pigtail output then was observed in real time using the transmittance spectra. By fine-tuning using a ball bearing vertical linear stage (Model MVN50, Newport Corporation, Irvine, CA, USA) and observing the corresponding transmittance spectra, precise alignment could be accomplished when the reading was tuned to the maximum value. The alignment used in this study is shown in Figure 3.

Once the multimode pigtail alignments were finished, these substrates were set to face each other. They were clipped at the end to prevent any movement. After clipping, the substrates were placed in the oven. The substrates were conditioned at 200 ${}^{\circ }$C for 1 h prior to the curing process. If the channel had leakage after the bonding process, UV glue was used to cover the area that leaked. The UV glue was applied at the side of the glass. The microfluidic was then exposed to UV light in an UV exposure machine for about 20 s to cure the UV glue. Finally, a channel with a height of 125 μm, width up to 0.2 cm and integrated with 30 μm microelectrode and fibre optic multimode pigtail was fabricated.

### 2.4. Experimental Setup

**Figure 4:**
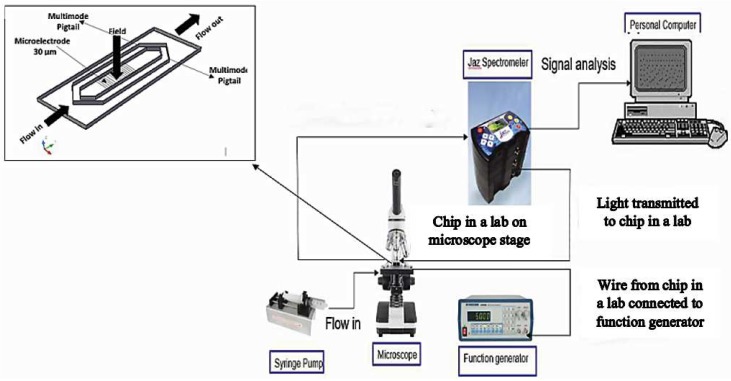
Experimental setup for integrated lab on chip.

The experimental setup for the chip-in-a-lab is shown in Figure 4. After sample preparation, a low concentration of bacterial and yeast cells with an OD of 0.3 and 0.5 respectively were inserted into the microfluidic using a syringe and a BASI pump (Model MD-1001, Bioanalytical Systems Inc., West Lafayette, IN, USA).

At the beginning of DEP-FFF separation, a 1 MHz signal with a voltage of 14 Vpk-pk was applied to the microelectrode using a waveform generator (Model 33500B Series, Agilent, Santa Clara, CA, USA). The cells were attracted to the microelectrode and photographed by the CCD camera. The cells that were not captured at the microelectrode were then flushed out to the optical detection channel. After 30 min, a pulsed DEP-FFF separation was carried out by using a waveform generator under continuous fluid flow. The pulsing times and flow rates were varied as shown in Table 1.

Afterwards, the sample was injected into the optical detection channel. The UV–Vis spectra were measured on a computer-controlled UV-Vis spectrometer (JAZ Spectrometer, Ocean Optic, Largo, FL, USA) using multimode pigtails as transmitter and receiver. The data was extracted and displayed on a personal computer.

## 3. Results and Discussion

### 3.1. Pulsed DEP-FFF Separation at Different Pulsing Times

To compare the difference between the rate of release and the percentage of cumulative absorbance, the separation of *E. coli* and *S. cerevisiae* were attempted using a pulse DEP-FFF technique in a separate experiment. In these experiments, the *E. coli* cells (OD600 of 0.3) and *S. cerevisiae* (OD600 of 0.5) were loaded into the channel. After appropriate voltage signals were applied to the microelectrodes, the cells would be attracted to the microelectrode by DEP forces.

#### 3.1.1. *Escherichia coli*

**Figure 5:**
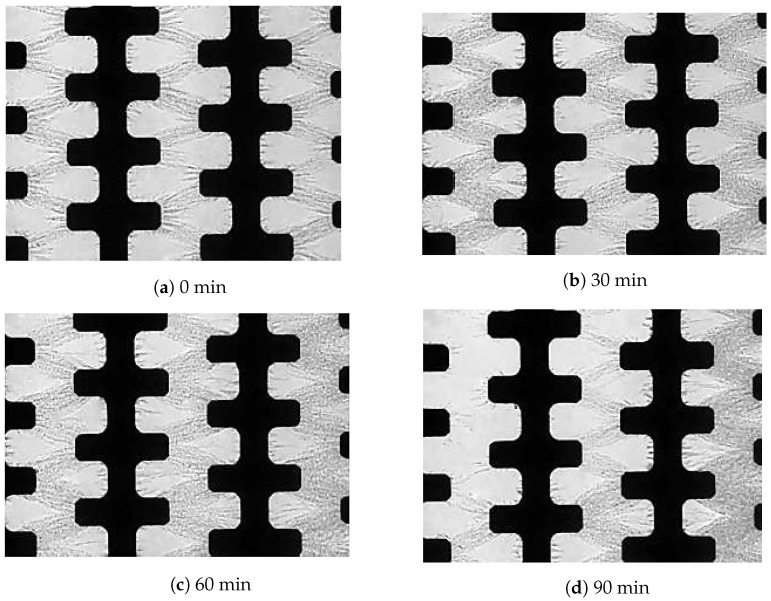
After 90 min of separation time, the *E. coli* concentration reduced significantly from the microelectrodes array 500 at a low conductivity σ${}_{m}$ of 40 μS/cm, pulsing time of 12 s and a high fluid flow of 9.6 μL min${}^{-1}$. The flow direction was from left to right.

Figure 5 presents the results obtained from the DEP-FFF experiment to separate *E. coli* under an AC electric field of 1 MHz and 14 Vpk-pk. At this conditions, *E. coli* cells travelled from the inlet and moved through the main channel by the effect of positive DEP and FFF as predicted [38,39].

At high frequency, low medium conductivity and high voltage, *E. coli* cells were strongly attracted to the microelectrode. In this work, the mechanism of the separation was achieved through pulsed DEP steps applied throughout the separation channel. During the application of AC electric field, the cells experienced DEP force, drag force, diffusion and gravitational force. All these forces acted upon the cells and produced a net forward effect to assist the movement of the cells towards the end of channel. On the other hand, when the electric field was switched off, the cell movement is determined only by the net effect from drag flow, gravity and diffusion. During this phase, FFF which is hydrodynamics plays the main role to control the cell movement or separation by controlling the dispersion and diffusion of the cells in the microfluidic channel. Hence, it is very important to determine the most optimum combination of time and flowrate that can result in a good separation.

Within 5–10 s after the exposure to the electric field, the cells were attracted to the microelectrode array 500 (refer to Figure 2), and fractionation was initiated (Figure 5a). Based on the colour contrast measurement, a reduced concentration of 10% *E. coli* cells were still attracted to the microelectrode after 90 min of separation time (Figure 5d). Two polarised particles come close to each other and undergo an attractive force to create a pearl chain. Normally a pearl chain is observed near an electrode edge where the strength of the electric field is the highest. The results obtained agreed with the previous work carried out by Suehiro and co-workers [40]. These results are due to the particle-particle interactions and therefore lead to being sterically trapped by other particle types surrounding them. These results match those observed in earlier studies [17].

#### 3.1.2. *Saccharomyces cerevisiae*

**Figure 6:**
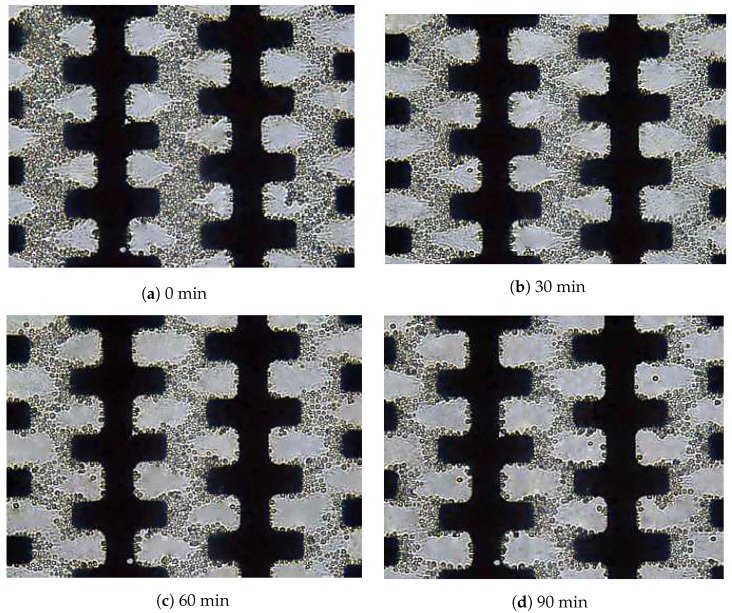
After 90 min of separation time, the *S. cerevisiae* concentration reduced gradually from the microelectrodes array 500 at a low conductivity σ${}_{m}$ of 40 μS/cm, a pulsing time of 12 s and a high fluid flow of 9.6 μL min${}^{-1}$. The flow direction was from left to right.

The experiment was performed using *S. cerevisiae* to determine their pulsing time-related behaviour. The distilled water was allowed to fill up and stabilised in the microchannel after about 5 min while the chosen flow rate was controlled by a syringe pump, as shown in Table 1. The concentrations of *S. cerevisiae* cells attracted to the microelectrode for a different pulsing time are shown in Figure 6. Again *S. cerevisiae* cells were attracted to microelectrodes and formed pearl chain formation as *E. coli* cells due to a positive DEP response as anticipated earlier and conformed to previous findings [41,42].

The result observed notable differences in DEP behaviour between *S. cerevisiae* and *E. coli*. For example, *S. cerevisiae* cells attracted to the microelectrode was progressively reduced as compared with *E. coli* cells response (compare Figure 6 with Figure 5). Only after 60 min of exposure to the high flow rate of 9.6 μL min${}^{-1}$ and a pulsing time of 12 s caused the *S. cerevisiae* cells to start to be released from the microelectrode. As can be seen in Figure 6d, most of the *S. cerevisiae* cells were still attracted to the microelectrode as compared to *E. coli* cells (compare Figure 6d and Figure 5d).

*E. coli* is a single-celled prokaryote of 1–3 μm in size that has no nucleus and membrane-bound organelles, while *S. cerevisiae* is a unicellular eukaryote of of 5–10 μm in size that has cell membrane and membrane bound organelles. With major difference in their cell structure and cell components, a difference in their dielectric properties is inevitable. At the application of electric field, several types of polarisation occur such as space-charge, orientation, ionic and electronics that arises from different parts of the cells structure or components (such as membrane morphologies, organelles, internal conductivities, and size) to give unique cell dielectric properties and behaviour [43]. This is in agreement with similar findings reported by other researchers [44,45,46]. Eventually, different dielectric properties resulted in different DEP and net forces acting on the cells that lead to different cell movement towards end of channel. Furthermore, the smaller size of *E. coli* cell facilitates it to travel faster and be eluted earlier based on FFF principles compared to *S. cerevisiae*.

### 3.2. Percentage of Cells Attracted to the Microelectrode Using Colour Contrast

Here the results are described based on the results obtained from the experiments in the DEP separation. From the previous experiment, the images of cells attracted to the microelectrode were captured by a CCD camera and analysed using the program ImageJ software. Image analysis was prepared according to the procedure used by other researchers [47]. The total area (pixels) of the cells attracted was measured and plotted on a graph. The graph was plotted as a percentage of cells retained at the microelectrode as a function of the time taken to release the cells from the microelectrode. The figures below show the result of the percentage of cells attracted to the microelectrode using colour contrast at the different pulsing time and flow rate.

#### 3.2.1. *Escherichia coli*

**Figure 7:**
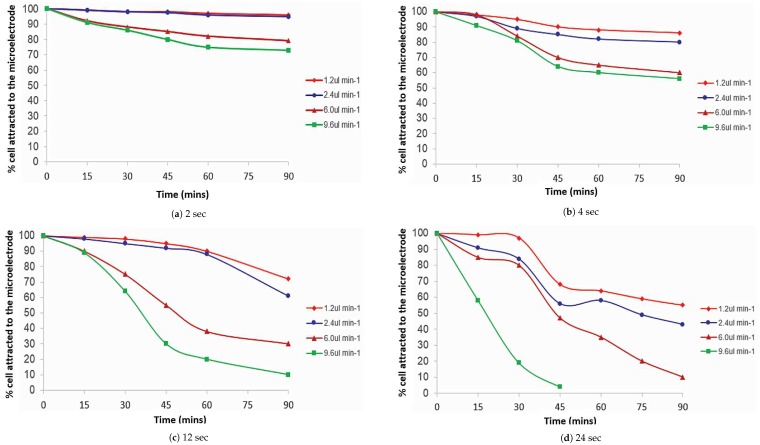
Percentage of *E. coli* cells attracted to the microelectrode as a function of time, comparing the pulsing time applied with the variation of flow rate.

Figure 7 presents the percentage of *E. coli* cells attracted to the microelectrode as a function of time, comparing the pulsing time applied with the variation of flow rate. Initially, Figure 7a shows a large amount of *E. coli* cells adhering to the surface of the microelectrode at a pulsing time of 2 s and a flow rate of 1.2 μL min${}^{-1}$. After 90 min, a gradual decrease in the number of cells attracted to the microelectrode was observed.

The figure also shows *E. coli* cells attracted to the microelectrode at a rapid pulsing time of 2 s and faster flow rate of 9.6 μL min${}^{-1}$. However, only 28% of the cells were released from the microelectrode after 90 min of separation time. The percentage of *E. coli* attracted to the microelectrode is given in Figure 7a where the use of different flow rates and a pulsing time of 24 s are shown. The figure shows that the cells attracted to the microelectrode was reduced when the flow rate was increased. The cells attracted to the microelectrode at a flow rate of 9.6 μL min${}^{-1}$ reduced by approximately 65% from the cells at the slow flow rate of 1.2 μL min${}^{-1}$.

#### 3.2.2. *Saccharomyces cerevisiae*

**Figure 8:**
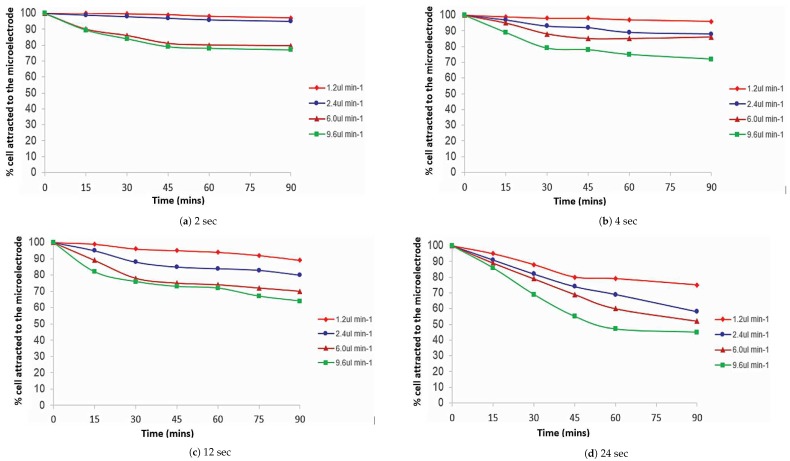
Percentage of *S. cerevisiae* cells attracted to the microelectrode as a function of time, comparing the pulsing time applied with the variation of flow rate.

Figure 8 shows the percentage of *S. cerevisiae* cells attracted to the microelectrodes with the variation of pulsing time and flow rates as a function of separation time. The results presented were obtained from the image analysis of the previous experiment.

In Figure 8a, the *S. cerevisiae* cells showed a similar trend to the *E. coli* cells for a pulsing time of 2 s. After 90 min, more than 90% of the cells still remained attracted to the microelectrode at a slower flow rate of 2.4 μL min${}^{-1}$. Nevertheless, at a faster flow rate (9.6 μL min${}^{-1}$) only 75% of the cells remained attracted to the microelectrode after 90 min of separation time. The percentage of cells attached versus separation time for a longer pulsing time (24 s) is shown in Figure 8d. It can be seen that 80% of cells were still attracted to the microelectrode at a flow rate of 1.2 μL min${}^{-1}$ after 90 min of separation time. At a higher flow rate (9.6 μL min${}^{-1}$), the number of cells attracted to the microelectrode was reduced and plateaued towards the end of the experiment.

### 3.3. Comparison of the Rate Release of *Escherichia coli* and *Saccharomyces cerevisiae* Cells from the Microelectrode

**Figure 9:**
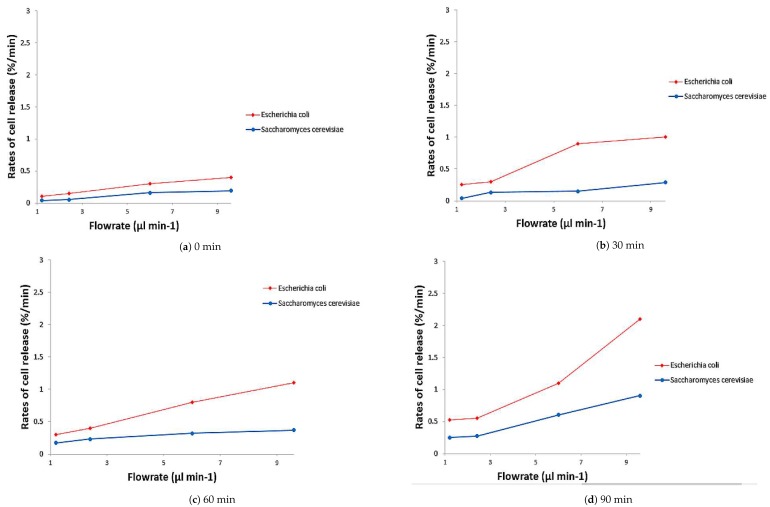
Comparison of the rate of release of *E. coli* and *S. cerevisiae* cells from the microelectrode at different flow rates and pulsing times.

The trends for comparison of the rate of release of *E. coli* and *S. cerevisiae* cells from the microelectrode are shown in Figure 9. There was little difference in the rate of release for both microorganisms at a pulsing time of 2 s as shown in Figure 9a. At this short pulsing time, both microorganisms were unable to achieve significant movement in the channel. The cells were seen to be collected again to the microelectrodes before it can move further away from its starting location.

With DEP-FFF applied, the rate of release of *E. coli* and *S. cerevisiae* cells increased slowly over the entire 90 min measurement duration (Figure 9a). More importantly, the rate of release of *E. coli* at around 0.3 %/min was 50% higher than that of *S. cerevisiae*. A similar pattern of rate of release was seen at a pulsing time of 12 s (Figure 9c). As evident from the figure, the rate of release demonstrated the highest difference rate of release at 0.6 %/min and *E. coli* exhibited almost double the rate of release compared to *S. cerevisiae* at a flow rate of 9.6 μL min${}^{-1}$. A different pattern was correlated between the flow rate and rate of cell release that was found when using a constant pulsing time of 24 s (Figure 9d). Interestingly, the results showed that the difference of the rate of cell release between two microorganisms increased dramatically 133% at the flow rate of 9.6 μL min${}^{-1}$. *E. coli* cells generally have a higher rate of release than *S. cerevisiae* cells, meaning that the *E. coli* cells could be eluted first from the microfluidic. This was a direct indication that the two microorganisms could be separated into essentially pure populations. In contrast, the rate of cell release for both microorganisms showed a significant difference at the pulsing time of 4 s, 12 s and 24 s.

### 3.4. Comparison of the Percentage of Cumulative Absorbance of *Escherichia coli* and *Saccharomyces cerevisiae* Cells

The experiment was performed on a suspension of mixed cells of *E. coli* and *S. cerevisiae*. A low concentration of *E. coli* (optical density of 0.3) and *S. cerevisiae* (optical density of 0.5) were introduced into the microfluidic. The corresponding concentrations were $8.0\times 10^{6}$ cells/mL and $8.6\times 10^{6}$ cells/mL for the *E. coli* and *S. cerevisiae* cells respectively. The *S. cerevisiae* cells were introduced first into the microfluidic and then followed by the *E. coli* cells. At the same time, the electric field at a frequency of 1 MHz with a voltage of 14 Vpk-pk was applied to separate these microorganisms. The DEP behaviour of the cells and the cumulative absorbance at a pulsing time of 12 s and a flow rate of 9.6 μL min${}^{-1}$ were recorded based on two wavelength range, which were 310 ± 10 nm for *E. coli* [48] and 435 ± 10 nm for *S. cerevisiae* [49].

**Figure 10:**
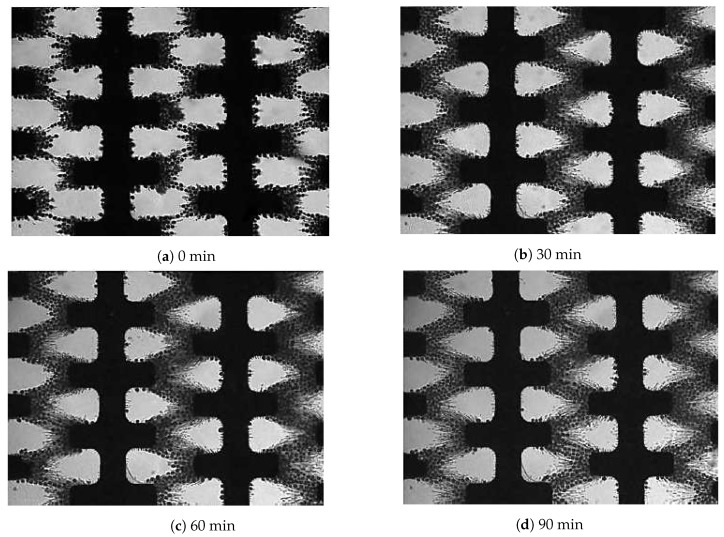
After 60 min of separation time, the mixture of *E. coli* and *S. cerevisiae* concentrations were still attracted to the microelectrodes at a low conductivity σ${}_{m}$ of 40 μS/cm, pulsing time of 12 s and a high fluid flow 9.6 μL min${}^{-1}$. The frequency applied was 1 MHz with a voltage of 14 Vpk-pk. The flow direction was from left to right.

Figure 10 shows the *S. cerevisiae* and *E. coli* cells attracted to the microelectrode. From this figure it can be seen that the *S. cerevisiae* and *E. coli* cells exhibited a positive DEP response at 1 MHz frequency and 14 Vpk-pk. It was apparent that the *S. cerevisiae* cells attracted more strongly to the microelectrode compared to the *E. coli* cells as explained earlier. Initially, the mixture of cells was attracted to the microelectrode immediately after the electric field was applied (Figure 10a). The cell concentration was found to increase gradually after 15 min (Figure 10b), followed by a continuous increase up until 60 min (Figure 10c,d). There were still more than 90% of the cells attracted to the microelectrode after 60 min of separation time.

**Figure 11:**
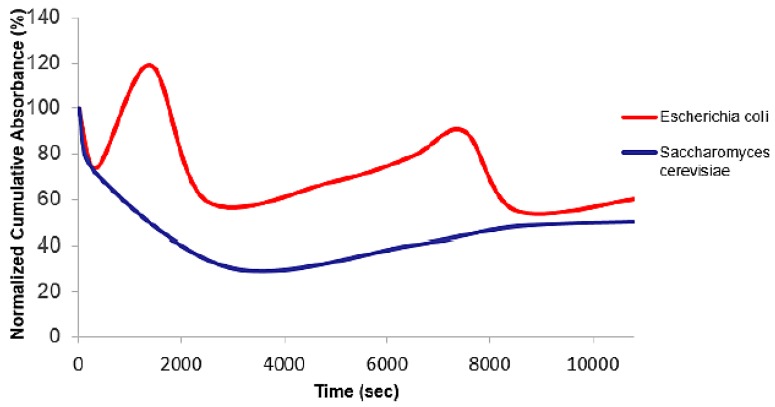
The percentage of cumulative absorbance of *E. coli* and *S. cerevisiae* cells that passed through the detection channel as a function of time at the pulsing time of 12 s applied with a flow rate of 9.6 μL min${}^{-1}$.

Figure 11 shows the percentage of cumulative absorbance of *E. coli* and *S. cerevisiae* cells that passed through the detection channel as a function of time. During the earlier phase of DEP-FFF separation process, many cells were attracted to the microelectrode while some of unattracted ones pass directly through to the fibre optic detection channel. At this instance, the absorbance measurement for that cell is low since it detects a small amount of cells in the detection channel. From the previous experiment, *E. coli* cells generally have a higher rate of release than the *S. cerevisiae* cells and tend to be eluted first from the channel (Figure 9). These experiments confirmed that *E. coli* cells released quickly from the microelectrode array and is subsequently detected with high absorbance value during the earlier period of experiments compared to *S. cerevisiae* cells. At the same time, *S. cerevisiae* cells have a low rate of release because the cells are attracted to the microelectrode array and hence should have a low absorbance value. The absorbance of both microorganisms rapidly decreased over the first few seconds until it reached 70% of the cumulative absorbance at 400 s. The cumulative absorbance of *E. coli* cells rose to 120% at 1000 s while the cumulative absorbance of the *S. cerevisiae* cells gradually dropped to nearly 30% until 3000 s of separation time. There was a sharp drop in the cumulative measurement for the *E. coli* cells until it reached 59% at 2500 s and this was followed by a more gradual increase over the next few minutes until 7500 s before decreasing again. The cumulative absorbance of the *S. cerevisiae* cells gradually increased to 43% until 10,000 s. This result suggests that the separation of microorganisms were more efficient when the difference between the percentages of cumulative absorbance was greater.

## 4. Discussion

These two microorganisms were chosen because of their difference in effective particle conductivity and cell size. The effective particle conductivity for *E. coli* and *S. cerevisiae* were 412 μS/cm and 16 μS/cm respectively [50]. Each cell was levitated at certain height because of the differences in the dielectric properties [51]. *S. cerevisiae* has a cell size of 8 to 10 μm in diameter while the cell size of *E. coli* was 1 to 2 μm in diameter. As can be seen, the *E. coli* cells were eluted first, and then the *S. cerevisiae* cells were eluted later. *S. cerevisiae* tends to be denser compared to *E. coli* and equilibrated nearer to the channel floor where the eluate flows slowly [52]. The *E. coli* cells were levitated higher into the faster moving regions of the eluate.

Furthermore, this result may be explained by the fact that *E. coli* and *S. cerevisiae* cells were different in shape and this led to the polarisation difference. These results appear to be consistent with other research [53]. When the pulsed DEP was applied, the polarisation discontinued and the charges changed to their original pattern of distribution. The time for the cells to revert to the original pattern of distribution depended on the kind of polarisation and cell size. The larger cell size had a slower change compared to the smaller cells [54].

In this investigation, it has been shown that two microorganisms can be isolated and detected using a chip-in-a-lab. This study successfully isolated the suspension of microorganisms such as *E. coli* and *S. cerevisiae* by exploiting the cell dielectric properties using the DEP-FFF method without the need for antibodies or other labelling procedures for separation and detection. These results were better than any reported separation because it was impractical to separate cells with similar sizes [55]. The most interesting finding was that it was possible to separate two nearly identical microorganisms in a suspension. The mechanism for separation of similar size microorganism demonstrated in a previous study involves a nano-orifice based DC-DEP microfluidic device [55]. An advantage of this method is that it uses only low electric potential applied locally. However, the method requires the adjustment of electric conductivity of the suspending medium and it is impractical in real-life applications.

The free space fibre optics approach appears promising in the detection of microorganisms. The study suggests that the highest separation between two microorganisms could be obtained by using a longer pulsing time of 12 s and a faster flow rate of 9.6 μL min${}^{-1}$ at a constant frequency, voltage and low conductivity. This was thought to be due to the different dielectric properties of the microorganisms, thus the microorganisms had a different rate of release. A UV spectrometer through a cumulative absorbance graph showed evidence that the separation occurred between the two microorganisms. Finally, this device provides a convenient method for possible applications of the biosensor.

## 5. Conclusions

The design and fabrication of a chip-in-a-lab device for separation and detection have been presented. A separation system has been designed to separate microorganisms in a lab on chip device using the pulsed field DEP-FFF mechanism. An optical system has been designed capable of detecting a microorganism moving within a device using light scattering. Chip in a lab allows for batch-mode experiments which are flexible and when staining or antibody modification was unnecessary, thus making the chip attractive in various applications. On the other hand, the development of integrated microsystems remains one of the most difficult challenges. The experimental results obtained in this study verified that different microorganisms could be simultaneously separated and detected using the chip-in-a-lab. The fabrication methods described in this paper could be used to further develop miniaturized particle diagnostic systems where detecting and/or sorting of cells is required. Future studies will focus on enhancing the separation time by reducing the number of microelectrode arrays and use a fibre insertion guide and mini lens to improve the performance of fibre optic detection.

## Figures and Tables

**Table 1:** Flow rates and pulsing times used in the experiments.

Flow Rate (μL/min)	Pulsing Time (s)
1.2	2
	4
	12
	24
2.4	2
	4
	12
	24
6.0	2
	4
	12
	24
9.6	2
	4
	12
	24

## Abbreviations

The following abbreviations are used in this manuscript:

DFR	Dry Film Resist
DEP-FFF	Dielectrophoresis-Field-Flow Fractionation
PDMS	Polydimethylsiloxane
